# Network Pharmacology and Molecular Docking of *Syzygium nervosum* Extracts on Antiproliferative Effect in Prostate Cancer

**DOI:** 10.3390/ijms27135977

**Published:** 2026-07-03

**Authors:** Napatsorn Saiyasit, Tanakamol Mahawan, Nitchakan Darai, Pilaiporn Thippraphan, Yawitthaphorn Soihin, Sunee Chansakaow, Aya Naiki-Ito, Satoru Takahashi, Weerakit Taychaworaditsakul

**Affiliations:** 1 Department of Medical Sciences, School of Medicine, Walailak University, Nakhon Si Thammarat 80160, Thailand; napatsorn.sa@wu.ac.th; 2 Center of Excellence in Tropical Pathobiology, Walailak University, Nakhon Si Thammarat 80160, Thailand; 3 Akkhraratchakumari Veterinary College, Walailak University, Nakhon Si Thammarat 80160, Thailand; m.tanakamol@gmail.com; 4 Research Center for Theoretical Simulation and Applied Research in Bioscience and Sensing, Walailak University, Nakhon Si Thammarat 80160, Thailand; nitchakan.da@wu.ac.th; 5 Futuristic Science Research Center, School of Science, Walailak University, Nakhon Si Thammarat 80160, Thailand; 6 Department of Biochemistry, Faculty of Medicine, Chiang Mai University, Chiang Mai 50200, Thailand; tipprapant@gmail.com (P.T.); yawitthaphorn@gmail.com (Y.S.); 7 Department of Pharmaceutical Sciences, Faculty of Pharmacy, Chiang Mai University, Chiang Mai 50200, Thailand; sunee.c@cmu.ac.th; 8 Department of Experimental Pathology and Tumor Biology, Graduate School of Medical Sciences, Nagoya City University, Nagoya 467-8601, Japan; ayaito@med.nagoya-cu.ac.jp (A.N.-I.); sattak@med.nagoya-cu.ac.jp (S.T.)

**Keywords:** prostate cancer, *Syzygium nervosum*, natural product, cytotoxicity

## Abstract

Prostate cancer (PCa) is one of the most common causes of cancer-related mortality in men globally. Although current therapies can control early-stage disease, advanced PCa remains difficult to treat because of therapeutic resistance and adverse side effects, highlighting the need for new treatment strategies. *Syzygium nervosum* (SN), a medicinal plant rich in bioactive compounds such as gallic acid and ellagic acid, has demonstrated anticancer properties in several malignancies; however, its effects on PCa remain unclear. This study investigated the anticancer potential of SN using integrated computational and in vitro approaches. DU145 and PC-3 prostate cancer cells were treated with SN extract at concentrations of 25–400 µg/mL for 24 and 48 h. Cell viability, colony formation, and cell-cycle progression were evaluated to determine antiproliferative activity. In parallel, computational analyses were performed to predict molecular targets of SN-derived compounds. Our results displayed that SN extract reduced cell viability, suppressed clonogenic growth, and disrupted cell-cycle progression in both cell lines. Computational findings suggested that gallic and ellagic acids may interact with key regulatory proteins related to cell proliferation and survival, including AKT and CDK2. Overall, SN demonstrates promising anticancer activity and may represent a potential therapeutic source for prostate cancer treatment.

## 1. Introduction

Prostate cancer (PCa) is a common cancer among men around the world. In 2022, an increasing prevalence of PCa occurred in men 45 years old or older, with an incidence rate of 119 per 100,000 men in the United States, as reported by the U.S. Centers for Disease Control and Prevention (CDC). The mortality of PCa has shown 3.8% of all deaths in 2018 [1]. In addition, the incidence of PCa has shown a consistent upward trend, with an increasing number of new cases reported in each year in Thailand [2]. PCa arises from the uncontrolled cell growth in the prostate gland. Even though the factors responsible for initiating prostate tumor development are still not well understood, several risk factors have been implicated, including advancing age, genetic predisposition, racial background, androgen levels (particularly testosterone), and environmental influences [3]. PCa presents across a spectrum of stages, ranging from localized disease confined to the prostate (early stage) to more aggressive forms that spread to other areas such as the lymph nodes or bones (late stage) [4]. In general, most PCa patients are diagnosed at a localized stage. However, once the event progresses to an aggressive or metastatic stage, it is associated with significantly poorer survival outcomes, with an estimated five-year survival rate of approximately 30% [5].

The progression of PCa is influenced by multiple interacting factors of cellular and molecular alterations that promote tumor growth, local invasion, and metastatic dissemination. PCa progression is closely linked to numerous factors, including oxidative stress, chronic inflammation, and the accumulation of genetic and epigenetic changes [6]. Among the various contributing factors, androgen signaling plays a central role in the initiation and development of the disease [7]. PCa is largely dependent on androgen receptor (AR) signaling in its early stages, with most tumors requiring androgen stimulation at diagnosis [7]. Androgen deprivation therapy (ADT), including surgical or chemical castration, represents the standard first-line treatment for advanced disease; however, despite initial responsiveness, most patients eventually progress to castration-resistant prostate cancer (CRPC) [7,8]. In CRPC, tumor growth continues despite reduced circulating androgen levels, which is mediated by AR amplification, AR mutations, intratumoral androgen synthesis, and activation of alternative survival pathway [7,9]. The management of PCa is guided by disease stage and clinical characteristics [10]. For CRPC, treatment options include next-generation AR pathway inhibitors (e.g., enzalutamide and abiraterone), taxane-based chemotherapy (docetaxel and cabazitaxel), radiopharmaceuticals, and other targeted agents [11]. However, the clinical efficacy of these therapies is often limited by acquired resistance, tumor heterogeneity, disease progression, and systemic toxicity. In addition, treatment-related adverse effects such as urinary dysfunction, erectile dysfunction, fatigue, nausea, and vomiting may significantly impair patient quality of life and restrict long-term treatment tolerance [10,12,13,14]. As a result, there is growing interest in alternative and complementary therapeutic strategies capable of targeting multiple oncogenic pathways simultaneously.

Natural products and plant-derived bioactive compounds have attracted attention due to their multi-targeted mechanisms, including modulation of cell-cycle regulation, induction of apoptosis, and inhibition of signaling pathways such as PI3K/AKT [15,16]. They have gained considerable attention in recent years for their promising roles in cancer therapy. Bioactive compounds derived from medicinal plants have demonstrated anti-cancer effects through various mechanisms, including the induction of apoptosis, inhibition of cell proliferation, suppression of metastasis, and modulation of oncogenic signaling pathways [17]. *Syzygium nervosum* (SN), or Ma-kiang, is a tropical medicinal plant commonly found in Southeast Asia, where it has long been used in traditional medicine. The leaves and flower buds are frequently prepared as herbal drinks and are traditionally associated with the treatment of respiratory, digestive, and skin-related disorders. A previous study has highlighted the plant as a rich source of bioactive compounds, including flavonoids, triterpenoids, and phloroglucinols, which may contribute to its reported antioxidant, anti-inflammatory, antidiabetic, and anticancer properties [18]. As previously mentioned, the predominant identified components of SN are gallic acid, pedunculagin, ellagic acid, and DMC (2′,4′-dihydroxy-6′-methoxy-3′,5′-dimethylchalcone) [19]. Studies have demonstrated the potential pharmacological activities of SN compounds in pathological-related mechanisms, including the attenuation of oxidative stress, anti-inflammation, and antitumor activity [18,20,21]. A component derived from SN extract was found to exert inhibiting tumor growth via the alleviation of cancer cell viability and proliferation. It also enhanced DNA damage, which contributed to elevating cancer cell death in several cancer cells, including human hepatoma, human colorectal, and human cervical cancer cells [19,22,23]. Together, these findings indicated that SN compounds are associated with anti-cancer effects. Although compounds from SN have been associated with anti-cancer activity, their cytotoxic effects and underlying molecular mechanisms in PCa remain unclear. Therefore, the current study aims to elucidate the target mechanisms of SN compounds in PCa using network pharmacology and molecular docking approaches and to investigate the cytotoxic effects of SN extract along with the related molecular mechanisms in human prostate cancer cells. This integrated strategy is expected to clarify the multi-target actions of SN and support its potential therapeutic application.

## 2. Results

### 2.1. Network Pharmacology of SN Compounds and Targeted Genes in PCa

**Figure 1:**
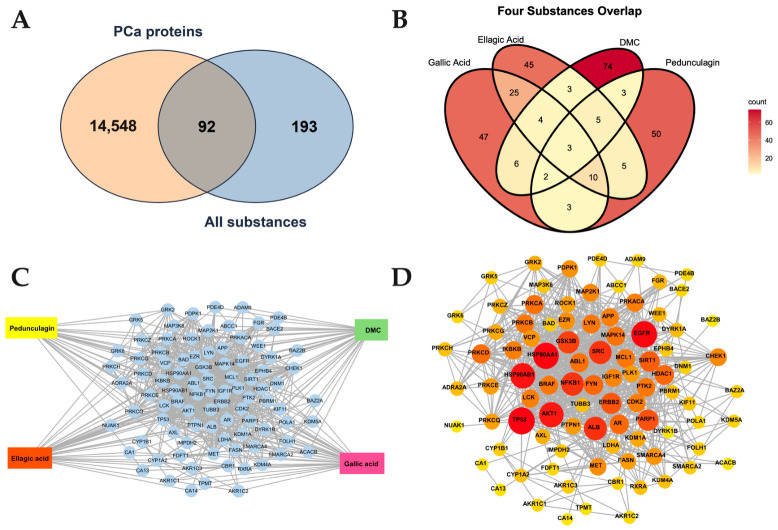
Network pharmacology analysis of SN compounds and their interactions with prostate cancer-related targets. (**A**) Venn diagram identifying 92 overlapping genes between PCa-associated proteins and compound targets. (**B**) Shared and unique targets among the four individual substances. (**C**) Compound-target network of the four main bioactive compounds. (**D**) Protein–protein interaction (PPI) network of all identified proteins visualized according to degree centrality. Node color represents degree centrality, with red indicating high degree, orange indicating moderate degree, and yellow indicating low degree. Node size is proportional to degree centrality (red = high, orange = moderate, yellow = low).

To elucidate the pharmacological mechanisms of SN compounds against PCa, target prediction and network analyses were performed. A total of 193 potential target genes were predicted for the four predominant identified compounds (gallic acid, pedunculagin, ellagic acid, and DMC). By intersecting these predicted targets with the acquired PCa proteomics dataset (comprising 14,548 background proteins), 92 overlapping target proteins were identified as potential therapeutic targets of SN compounds in PCa (Figure 1A). Further analysis revealed the distribution of these targets among the individual SN compounds. Specifically, the four-way Venn diagram (Figure 1B) demonstrates the unique and shared targets among the substances, highlighting a core set of five genes concurrently targeted by all four compounds.

To visualize the systemic interactions, a bipartite compound-target network was constructed (Figure 1C). Subsequently, the 92 overlapping proteins were mapped using the STRING database to generate a protein–protein interaction (PPI) network. Topological analysis utilizing the cytoHubba plugin identified key hub genes within this network (Figure 1D). Based on degree centrality, the highest-ranking core targets (highlighted in red) included critical cancer-associated genes such as TP53, EGFR, AKT1, SRC, HSP90AA1, and HSP90AB1, suggesting their central role in the therapeutic action of SN compounds against PCa.

### 2.2. Gene Ontology (GO) and KEGG Pathway Enrichment

**Figure 2:**
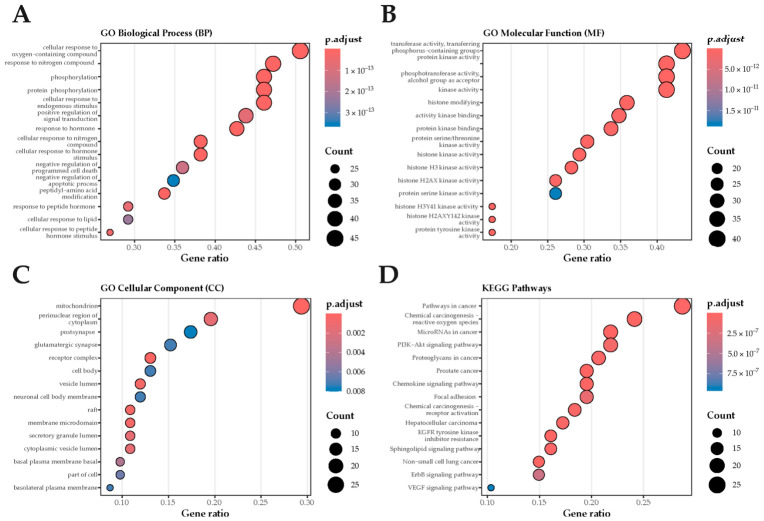
Functional and pathway enrichment analysis of the 92 overlapping target genes. Dot plots illustrating the top significantly enriched categories for (**A**) GO Biological Process (BP), (**B**) GO Molecular Function (MF), (**C**) GO Cellular Component (CC), and (**D**) KEGG pathways. The size of the dots represents the gene. Bubble size represents gene count; color indicates adjusted *p*-value. Gene ratio on x-axis shows the proportion of enriched genes.

To further investigate the biological functions and molecular mechanisms of the 92 intersecting target genes, GO and KEGG pathway enrichment analyses were performed using the *clusterProfiler* R package. For GO analysis, the target genes were significantly enriched in various Biological Processes (BP), primarily involving the cellular response to oxygen-containing compounds, phosphorylation, and protein phosphorylation (Figure 2A). Regarding Molecular Function (MF), the targets were strongly associated with kinase activities, notably transferase activity, transferring phosphorus-containing groups, protein kinase activity, and phosphotransferase activity (Figure 2B). Cellular Component (CC) analysis indicated that these proteins are predominantly localized to the mitochondrion, perinuclear region of cytoplasm, and postsynapse (Figure 2C). Moreover, KEGG enrichment analysis revealed that the target genes were significantly implicated in vital oncogenic and signaling pathways (Figure 2D). The most highly enriched pathways included broad categories such as pathways in cancer, chemical carcinogenesis—reactive oxygen species, microRNAs in cancer, and the PI3K-Akt signaling pathway. Notably, the specific PCa pathway was also significantly enriched, directly supporting the therapeutic potential of SN compounds in targeting PCa-specific disease mechanisms.

### 2.3. Cytotoxicity in Human Prostate and Normal Cells Treated with SN Extract

**Figure 3:**
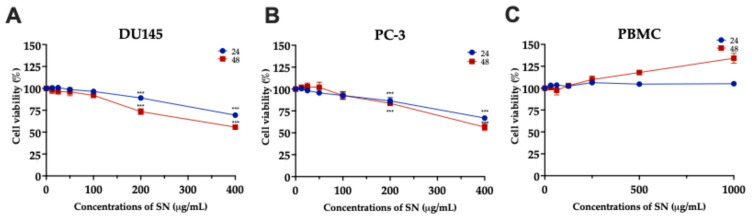
Cytotoxicity of SN extract in prostate cancer cells and normal immune cells. (**A**) DU145, (**B**) PC-3, and (**C**) PBMC cells were exposed to varying concentrations of SN extract for 24 and 48 h, followed by SRB assay. The concentration of SN for DU145 and PC3 cells are varying at 12.5, 25, 50, 100, 200, and 400 µg/mL. SN Doses for PBMC cell are 31.35, 62.5, 125, 250, 500, and 1000 µg/mL. Data are expressed as mean ± SD from three independent experiments. Statistical significance vs. control: *** *p* < 0.001.

As illustrated in Figure 3, the SRB assay was used in this study to assess cytotoxicity in DU145, PC-3, and PBMC cells exposed to SN at different doses for 24 and 48 h. SN treatment significantly reduced cell viability in both DU145 and PC-3 cells in a dose- and time-dependent manner, especially at 200 and 400 µg/mL (Figure 3A,B). Notably, SN showed no cytotoxicity toward PBMC cells even at concentrations exceeding 1000 µg/mL, indicating excellent safety toward normal cells (Figure 3C). The IC_20_ and IC_50_ values for each cell line are presented in Table 1. To evaluate the selectivity of SN toward prostate cancer cells, the selectivity index (SI) was calculated as shown in Table 1. The selectivity indices were >1.59 at 24 h and >2.15 at 48 h, confirming that SN selectively targets prostate cancer cells. These findings suggest that SN has therapeutic potential as a selective anticancer agent against human prostate cancer cells.

### 2.4. Effect of SN Treatment on Colony Formation

**Figure 4:**
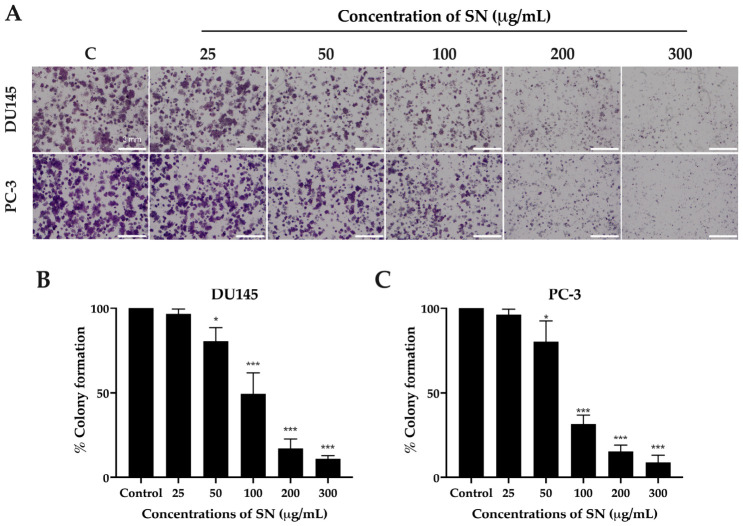
Inhibition of colony formation by SN extract in PCa cells. (**A**) Representative microscopic images of colony formation assay showing crystal violet-stained colonies of DU145 (**upper panel**) and PC-3 (**lower panel**) cells treated with various concentrations of SN extract or vehicle control for 10 days (scale bar = 3000 μm, 4× objective). Quantitative analysis of (**B**) DU145 and (**C**) PC-3 colony formation expressed as a percentage relative to untreated control cells. Data are expressed as mean ± SD from three independent experiments. Statistical significance: * *p* < 0.05 and *** *p* < 0.0001 compared to control group.

To examine the antiproliferative effect of SN extract, colony formation assay was conducted. This experiment used SN extract values below IC_20_ at various dosages in DU145 and PC-3 cells after 48 h. Representative images and quantitative data on colony formation are shown in Figure 4A. The percentage of colony formation was decreased in a concentration-dependent manner in both cells, compared with control of each cell type (Figure 4B,C). A reduction in colony formation was observed at 50 μg/mL, with the greatest decrease at 300 μg/mL (Figure 4B,C). Thereby, SN treatment for 48 h exerts the long-term antiproliferative effect in PCa cells.

### 2.5. Effect of SN Treatment on Cell Cycle

**Figure 5:**
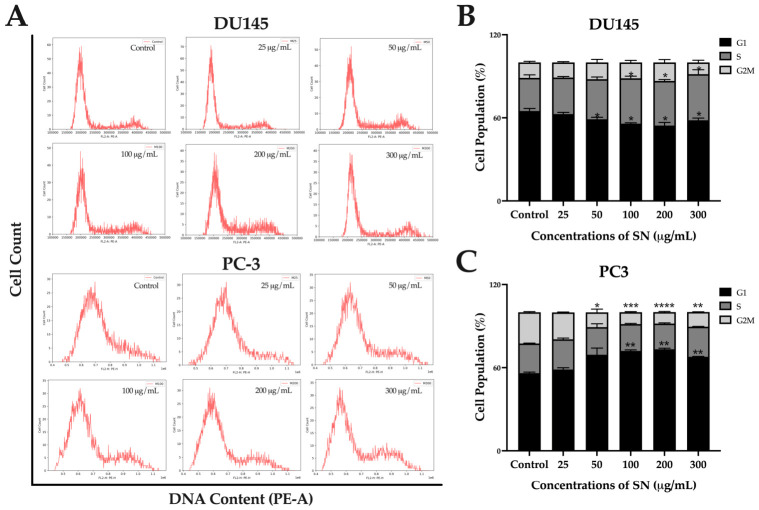
SN extract-induced cell cycle arrest in prostate cancer cells. (**A**) Representative flow cytometry histograms of DNA content in DU145 and PC-3 cells after SN treatment for 48 h. (**B**,**C**) Cell cycle phase distribution showing percentage of cells in G1, S, and G2/M phases in DU145 and PC-3 cells. Data are expressed as mean ± SD from three independent experiments (*n* = 3). Statistical significance: * *p* < 0.05, ** *p* < 0.01, *** *p* < 0.001, and **** *p* < 0.0001 compared to control group.

**Figure 6:**
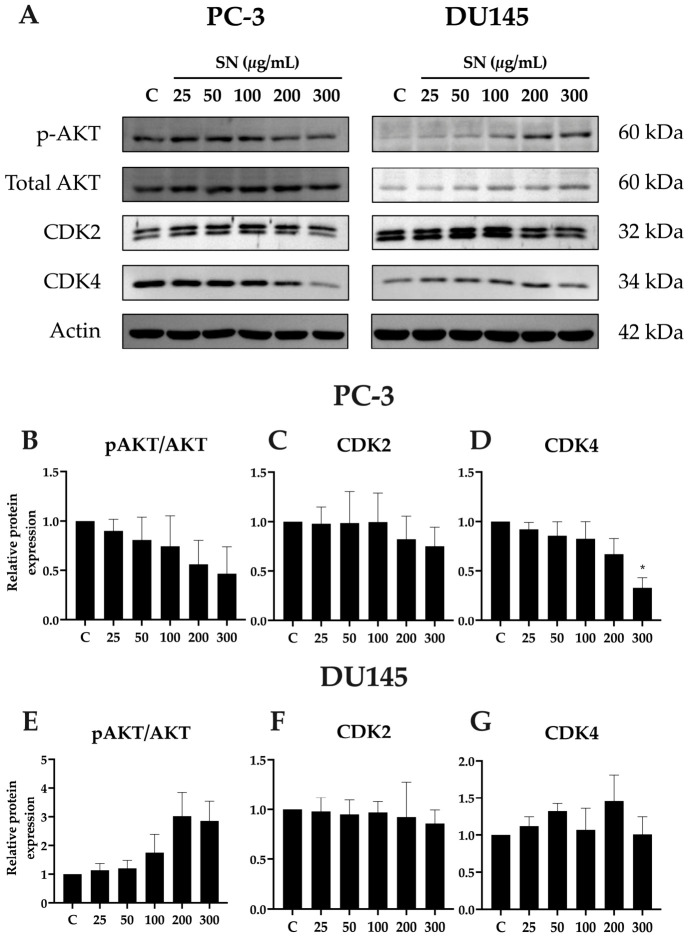
Potential effects of SN extract on cell cycle regulatory proteins. (**A**) Representative Western blot images showing expression of p-AKT, total AKT, CDK2, and CDK4 in PC-3 and DU145 cells treated with SN extract (0–300 µg/mL) for 48 h. (**B**–**D**) Quantification of protein expression in PC-3 cells showing relative levels of p-AKT/AKT ratio, CDK2, and CDK4. (**E**–**G**) Quantification of protein expression in DU145 cells. Data normalized to actin and are expressed as mean ± SD from three independent experiments (*n* = 3). Statistical significance: * *p* < 0.05 compared to control group.

Flow cytometric analysis was used to explore the effect of SN extract on cell cycle distribution in DU145 and PC-3 cells, as displayed in Figure 5. In DU145 cells, treating with SN extract contributed to a significant decrease in the proportion of cells in the G1 phase, accompanied by a concurrent elevation in the S phase population, especially at concentrations of 100–300 µg/mL (Figure 5B). At the same concentration in PC-3 cells, SN extract elevated the G1 phase and reduced the G2/M phase (Figure 5C). Furthermore, changes in the expression of proteins related to the cell cycle are consistent with these findings. SN extract decreased CDK4 protein levels and showed a trend toward reducing p-AKT levels in PC-3 cells, with the most significant reduction observed at 300 µg/mL. Nevertheless, p-AKT showed an increasing trend at higher concentrations, particularly at 200–300 µg/mL, while CDK4 protein levels remained relatively unchanged in DU145 cells. CDK2 expression demonstrated a decreasing trend in both PC-3 and DU145 cells. However, the drop did not attain statistical significance (Figure 6). Overall, these findings suggest that SN extract induces cell cycle arrest in both cell lines, but through distinct phase-specific regulatory mechanisms.

### 2.6. Molecular Docking of Gallic Acid and Proliferative-Related Molecules

**Figure 7:**
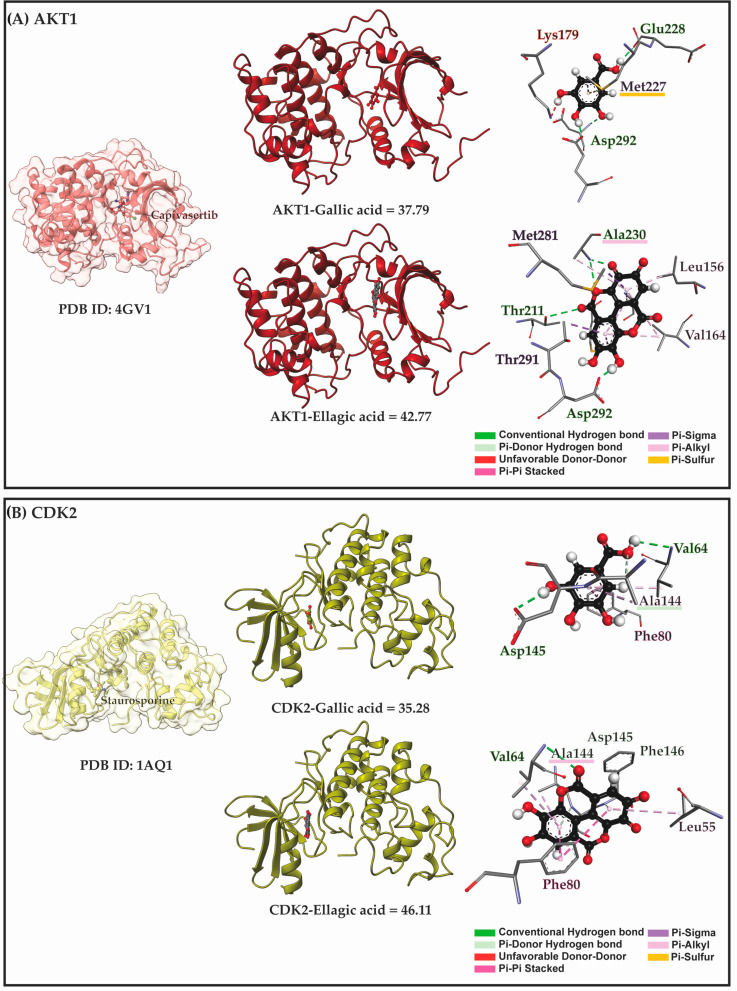
Molecular docking analysis of gallic acid and ellagic acid with proliferation-related protein targets. (**A**) Docking poses and interaction profiles of gallic acid and ellagic acid within the AKT1 binding pocket using the crystal structure of AKT1 complexed with the co-crystallized ligand capivasertib (PDB ID: 4GV1). (**B**) Docking poses and interaction profiles of gallic acid and ellagic acid within the CDK2 binding pocket using the crystal structure of CDK2 complexed with the co-crystallized ligand staurosporine (PDB ID: 1AQ1).

Following the network pharmacology analysis and in vitro experiments, molecular docking was subsequently performed to further evaluate the potential molecular interactions between SN compounds (gallic and ellagic acids) and selected proliferation-related protein targets. Two cancer-associated proteins, AKT1 and CDK2 (Figure 7A,B), were selected for docking analysis because of their important roles in cancer-related signaling pathways, including cell proliferation, apoptosis, cell-cycle regulation, and metastasis. The crystal structures of AKT1 and CDK2 were obtained from the Protein Data Bank using PDB IDs 4GV1 and 1AQ1, respectively. The co-crystallized ligands, capivasertib in AKT1 and staurosporine in CDK2, were used as reference ligands to define the active binding pockets for docking. Furthermore, the redocking validation of docking protocol was demonstrated in Supplementary Materials (Figure S1).

Gallic acid and ellagic acid were selected as docking ligands because both compounds were previously identified in the SN extract. The docking results showed that both compounds could be accommodated within the binding pockets of AKT1 and CDK2. The docking values are reported as GOLD fitness scores, where higher positive scores indicate better predicted ligand-protein binding fitness rather than binding free energy. The docking pose of gallic acid within the AKT1 binding pocket revealed interactions with Lys179, Met227, Glu228, and Asp292. Gallic acid formed conventional hydrogen bonds with Glu228 and Asp292, which may play an important role in stabilizing the ligand within the active site. Additional π-sigma and π-sulfur interactions were observed with Met227, suggesting further contribution to ligand–protein binding. Although an unfavorable donor-donor interaction was detected with Lys179, the overall interaction profile indicates that gallic acid can bind favorably within the AKT1 active pocket. For CDK2, gallic acid formed multiple interactions with key amino acid residues within the binding pocket, including Val64, Phe80, Ala144, and Asp145. Conventional hydrogen bonds were observed with Val64 and Asp145, which may contribute to binding specificity and stabilize gallic acid within the active site. In addition, Phe80 was involved in a π–π stacked interaction, indicating that the aromatic ring of gallic acid may interact favorably with the aromatic side chain of Phe80. Ala144 contributed through π-alkyl and π-donor hydrogen bond interactions, which may further stabilize the ligand in the hydrophobic region of the binding pocket. Overall, the docking results suggest that gallic acid has the potential to interact with both AKT1 and CDK2, with a stronger predicted binding preference toward AKT1.

In addition to gallic acid, ellagic acid was also docked with AKT1 and CDK2 because it was identified in the SN extract at higher abundance as shown in Figure 7. Ellagic acid showed higher GOLD fitness scores than gallic acid for both targets, with scores of 42.77 for AKT1 and 46.11 for CDK2. Within the AKT1 binding pocket, ellagic acid interacted with Leu156, Val164, Thr211, Ala230, Met281, Thr291, and Asp292. Leu156 and Val164 contributed through π-alkyl interactions, while Met281 and Thr291 were involved in π-sigma interactions. In addition, Thr211, Ala230, and Asp292 formed hydrogen bonds with ellagic acid, which may help stabilize the ligand within the AKT1 active site. For CDK2, ellagic acid interacted with Leu55, Val64, Phe80, Ala144, Asp145, and Phe146. Leu55 and Ala144 contributed through π-alkyl interactions, Val64 formed a conventional hydrogen bond, and Phe80 was involved in a π–π stacked interaction. Additional π-donor hydrogen bond interactions with Asp145 and Phe146 may further support the stable accommodation of ellagic acid within the CDK2 binding pocket. These findings suggest that ellagic acid may also contribute to the predicted interactions of the SN extract with proliferation-related targets.

## 3. Discussion

This study integrated network pharmacology and molecular docking to identify potential targets of SN compounds and to explore their mechanisms of action against PCa. SN extract exhibited antiproliferative effects in PCa cells, as shown by reduced cell viability, diminished colony formation, and disrupted cell-cycle progression. These effects were supported by computational analyses, which indicated that key SN bioactive compounds, specifically gallic acid, may interact with AKT and CDK2, proteins essential for cell survival and proliferation.

In this study, the four compounds were selected for network pharmacology analysis because previous phytochemical studies identified them as the predominant characterized constituents of the SN extract, whereas the remaining compounds were unidentified [19]. Future studies should characterize these unidentified constituents to provide a more comprehensive understanding of the pharmacological properties of the SN extract. Our network pharmacology analysis revealed that bioactive compounds of SN extract potentially interact with key PCa proteins, especially AKT, a central regulator of cell proliferation, survival, and metabolic signaling [24]. AKT is tightly controlled by multiple upstream regulators and interacting proteins, including phosphoinositide 3-kinase (PI3K), phosphatase and tensin homolog (PTEN), and receptor tyrosine kinases, which collectively determine its activation status and downstream signaling output [15,24,25,26]. Dysregulation of the PI3K/AKT signaling pathway contributes to tumor initiation, progression, therapeutic resistance, and hormone-independent (androgen-independent) prostate cancer [24,25,26]. In the present study, DU145 and PC-3 cells, as both represent established models of androgen-independent PCa with distinct alterations in PI3K/AKT signaling, were used. PC-3 cells are characterized by PTEN loss, resulting in constitutive AKT pathway activation, whereas DU145 cells exhibit alternative regulatory defects that also contribute to dysregulated AKT signaling and impaired checkpoint control [27,28,29]. These molecular features make both cell lines highly suitable for evaluating agents targeting the AKT pathway in PCa. Therefore, the predicted interaction of SN compounds with AKT provides mechanistic support for the observed antiproliferative effects in hormone-refractory prostate cancer.

Consistent with the predicted molecular targets, SN extract reduced cell viability while selectively affecting cancer cells without toxicity to PBMCs, indicating selective anticancer activity. Supporting this finding, previous studies have reported SI values ranging from 1.7 to 4 for extracts from other *Syzygium* species, depending on the exposure duration [19], which corresponds to moderate to strong anticancer potential [30,31]. Excitingly, higher concentrations of SN extract increased PBMC proliferation, suggesting possible immunostimulatory effects. Similar immune-enhancing activities of flavonoids from other *Syzygium* species have been stated in relation to PBMC responses and cytokine production [32,33]. Thereby, the changes observed in PBMCs may indicate a possible immunomodulatory effect; however, without specific immune function assays, this interpretation remains preliminary and should be considered a hypothesis that requires further experimental confirmation. Together, these findings suggest a dual mechanism involving cytotoxic effects on cancer cells and stimulation of immune responses, which may lead to tumor control. Further studies are required to elucidate the immunomodulatory mechanisms, including cytokine signaling and lymphocyte activation.

In addition, the reduced colony formation further shows a long-term suppression of proliferative capacity, indicating that SN extract impairs cancer cell growth and survival over time. These findings align with previous reports showing that natural polyphenolic compounds possess antiproliferative and apoptotic properties in various cancer models [19,34,35,36,37]. For instance, using crude extracts derived from *Syzygium* species, including SN and *Syzygium aromaticum* (SA), exhibited anticancer activity by suppressing cell proliferation and promoting apoptosis in colorectal cancer cells [19]. These effects may be associated with the presence of phenolic constituents, particularly gallic acid and ellagic acids, which are commonly found in both plants [38]. Evidence from studies using isolated compounds further supports this assumption. Treatment with gallic acid inhibited growth and proliferation while inducing apoptosis in DU145 and human cervical cancer cells [37,39]. The aforementioned findings indicate that phenolic constituents are likely important contributors to the antiproliferative and apoptotic properties observed in *Syzygium* extracts. Apoptosis was not assessed in the present study; however, previous studies have demonstrated that SN extract can induce programmed cell death in a range of cancer cell types [22,40]. Accordingly, further investigations are warranted to determine whether apoptotic pathways also contribute to its effects in prostate cancer models.

Notably, SN extract interferes with cell-cycle control in a cell line-specific manner by targeting distinct CDK-regulated checkpoints. In DU145 cells, the impairment of G1/S progression and induction of replication stress is consistent with slightly reduced activity of CDK2, which normally partners with cyclins E and A to drive DNA replication and S-phase entry [41]. In PC-3 cells, the accumulation in G1 and reduction of G2/M transition align with decreased CDK4 function, a key regulator of early G1 progression over cyclin D-dependent phosphorylation of retinoblastoma protein [42]. The abovementioned results indicate that SN extract suppresses proliferation by disrupting different CDK-mediated control points in the cell cycle depending on the cellular context. These findings are consistent with previous reports on polyphenolic compounds in cancer models [36,43,44,45]. Ellagic acid-rich compounds have been shown to induce S-phase arrest and apoptosis in PCa cells [43], while gallic acid can trigger phase-specific cell-cycle arrest depending on cellular conditions, including in colorectal, breast, and prostate cancer cells [36,44,45]. Additionally, our findings show that SN extract produced more pronounced alterations in cell-cycle distribution and viability in DU145 cells than PC-3 cells, which is consistent with the distinct molecular backgrounds of these two androgen-independent prostate cancer models [46,47,48]. DU145 cells harbor a mutant p53 but retain PTEN expression, resulting in comparatively lower basal AKT activity and a more preserved balance between pro-survival and pro-apoptotic signaling [47,48]. In contrast, PC-3 cells are PTEN-null and p53-deficient, with constitutively hyperactivated PI3K/AKT signaling that supports a highly aggressive, apoptosis-resistant phenotype and reduced sensitivity to a variety of cytotoxic and targeted agents [46]. Within this context, the greater responsiveness of DU145 to SN-induced modulation of CDK-regulated checkpoints and AKT-linked pathways may reflect its lower dependence on PI3K/AKT hyperactivation and its partially retained checkpoint control, whereas the more attenuated effects observed in PC-3 likely mirror its stronger reliance on PTEN loss-driven AKT signaling and intrinsic therapy resistance [49]. Nevertheless, further mechanistic work is needed to define its broader signaling impact, including upstream PI3K components, downstream effectors such as mTOR, and compensatory pathways like MAPK. Extending these studies to additional prostate cancer models, including androgen-dependent and -independent lines such as LNCaP and 22Rv1, would also help clarify whether its activity is influenced by androgen receptor status and tumor subtype. Overall, SN extract inhibited cell viability, colony formation, and cell-cycle progression in the current study. However, it did not significantly suppress p-Akt or CDK2 expression in either DU145 or PC-3 cells and even increased p-Akt levels at 200–300 μg/mL in DU145 cells. These findings suggest that the observed anticancer effects may involve alternative signaling pathways, which should be further investigated. Nonetheless, the anticancer activity of *Syzygium* crude extracts is likely driven by the combined and possibly synergistic effects of multiple phytochemicals rather than a single active compound. Therefore, further mechanistic studies are needed to clarify these interactions and identify the key bioactive constituents, together with in vivo validation to better evaluate their therapeutic potential.

Finally, molecular docking analysis was performed to investigate how SN compounds may influence AKT and CDK2 activity, based on the aforementioned in silico and in vitro findings. AKT was selected due to its central role in the PI3K/AKT signaling pathway involved in cell proliferation, whereas CDK2 was included based on its identification as a key hub protein in the PPI network and its established role in cell-cycle regulation. Although no significant change in p-AKT levels was observed experimentally, this may reflect context-dependent or cell line-specific regulation of AKT signaling. In addition, other hub proteins identified from the PPI network, particularly those associated with tumor suppression and apoptosis, such as TP53 and EGFR, may also contribute to the observed biological effects and should be explored in future studies. Our results demonstrated that gallic and ellagic acids, identified as the predominant identified constituents of the extract, were selected due to their reported anti-proliferative properties. The docking results suggest that both gallic and ellagic acids can bind favorably to both AKT and CDK2, with affinities comparable to known inhibitors, indicating a potential for direct target interaction. This is consistent with previous experimental evidence showing that gallic acid can suppress AKT activation and reduce its phosphorylation, leading to inhibition of downstream survival signaling and decreased cancer cell proliferation [50,51]. Similarly, it has been reported to downregulate CDK2 expression and disrupt cyclin-CDK complex activity, thereby impairing cell-cycle progression and promoting apoptosis [52,53]. Ellagic acid reduced the expression of p-AKT and CDK2 related to the inhibition of pancreatic cancer cell growth [54]. Moreover, reduced CDK2 expression has been associated with suppressed AKT signaling, suggesting coordinated regulation of cell cycle progression by the AKT/CDK2 axis [55]. Overall, these findings support a multi-target mechanism in which gallic acid and ellagic acids may simultaneously attenuate AKT-driven survival pathways and CDK2-mediated cell-cycle regulation, contributing to reduced tumor growth and enhanced cell-cycle arrest. However, AKT/CDK2 involvement remains a prediction supported by preliminary experimental observations. Therefore, further experimental validation, such as enzyme inhibition assays or target-based cellular assays, is required to confirm the predicted molecular docking results.

To describe the interaction between SN structural features and their biological activities, the four predominant SN constituents display complementary structural motifs that appear consistent with, and help rationalize, their predicted interactions. Gallic acid is a small 3,4,5-trihydroxybenzoic acid whose single aromatic ring, dense hydroxylation, and free carboxylate create a compact, hydrogen bond-rich surface that fits well into the ATP-binding pockets of AKT1 and CDK2 and supports multiple polar contacts, in line with our docking results. Ellagic acid extends this design into a rigid, planar biaryl dilactone with four phenolic hydroxyl groups and an enlarged π-conjugated system, yielding docked poses within AKT1 and CDK2 that stably engage key active-site residues through combined hydrogen-bonding and π–π interactions. Although gallic acid and ellagic acid possess a similar capacity for hydrogen-bond donation through their hydroxyl groups, the dilactone scaffold of ellagic acid introduces additional carbonyl oxygen atoms that can serve as hydrogen-bond acceptors. Together with its larger and more rigid aromatic framework, these structural features provide increased opportunities for productive interactions within the ATP-binding pockets of AKT1 and CDK2. Consistent with this observation, ellagic acid exhibited more favorable binding scores than gallic acid in our docking analysis, suggesting stronger binding affinity and greater stabilization of the protein–ligand complexes. The increased number of conventional hydrogen-bond interactions, together with enhanced π-mediated contacts, may therefore contribute to the potentially greater biological activity of ellagic acid relative to gallic acid. Overall, the docking results support the notion that the structural characteristics of these polyphenols are closely associated with their ability to interact with cancer-related molecular targets and may underlie their reported antiproliferative properties.

Consistent with these structural and in silico observations, gallic acid, a predominant identified constituent of SN, has been reported to exert antiproliferative and pro-apoptotic effects in various cancer models through ROS generation, cell-cycle arrest, and modulation of the PI3K/AKT signaling pathway [56,57,58,59]. Likewise, ellagic acid exhibits selective antiproliferative activity and induces apoptosis in several tumor cell lines, including DU145 prostate cancer cells, further supporting the contribution of this polyphenolic scaffold to the overall anticancer potential of SN [60]. Pedunculagin, a highly hydroxylated ellagitannin, has also demonstrated antitumor and cytotoxic activities against human breast cancer cells [61], whereas the chalcone DMC from SN exerts potent antiproliferative effects through induction of DNA damage, G0/G1 cell-cycle arrest, and apoptosis in human cervical cancer cells [22]. Taken together, the favorable interactions observed in the molecular docking analysis, coupled with the documented anticancer activities of the individual constituents, suggest that the antiproliferative effects of SN extract are likely mediated through the complementary and potentially synergistic actions of its major polyphenolic compounds rather than a single bioactive constituent. Nevertheless, the contribution of minor phytochemicals and the precise mechanisms underlying these combined effects remain to be elucidated through further mechanistic and in vivo studies.

Taken together, the integration of computational and experimental findings suggests that SN exerts its anticancer effects involving modulation of AKT signaling and disruption of CDK-regulated cell cycle progression. However, further investigations are required to clarify its precise mechanisms of action, as well as to validate its efficacy and safety in vivo and in clinical settings. Future studies should also address pharmacokinetic properties, optimal dosing strategies, and potential interactions with existing therapies to support the development of SN extract as an alternative or complementary therapeutic option for prostate cancer.

## 4. Materials and Methods

### 4.1. Preparation of SN Extract

SN extraction was obtained from Assistant Professor Dr. Sunee Chansakaow, Faculty of Pharmacy at Chiang Mai University. The method for SN extraction and the identification of SN components using LC-DAD-Q-Orbitrap-MS/MS was described in a previous experiment [19].

### 4.2. In Silico Experiments

#### 4.2.1. Identification of Bioactive Compounds, Target Prediction, and Acquisition of PCa Proteomics Data

The predominant identified phytochemical constituents of SN extract, specifically Gallic acid, Pedunculagin, Ellagic acid, and 2′,4′-Dihydroxy-6′-methoxy-3′,5′-dimethylchalcone (DMC) as previously reported [19], were prioritized for network pharmacology analysis. Canonical SMILES strings and PubChem CID for each substance were retrieved from the PubChem database. Potential macromolecular targets were predicted using the SwissTargetPrediction platform (http://www.swisstargetprediction.ch/; accessed on 5 February 2026). SMILES strings were queried against the Homo sapiens dataset, and proteins with high probability scores were retained. All predicted targets were cross-referenced with the UniProtKB database to standardize entry names, official gene symbols, and biological functions.

To identify proteins associated with PCa progression, we utilized high-resolution proteomics data with clinical relevance to advanced disease. Following a systematic review of available resources, the phosphoproteomics dataset was selected due to its comprehensive coverage (~8300 phosphopeptides) and inclusion of metastatic castration-resistant prostate cancer (CRPC) samples [62]. Data retrieval was performed in R (v4.5.1) using the rpx (v2.16.0) package. The dataset was accessed via ProteomeXchange (accession: PXD002286). Raw spectra and processed identification tables were downloaded to extract protein profiles associated with invasive/advanced disease features for integrative network analysis.

#### 4.2.2. Network Construction and Topology Analysis

A multilayer network pharmacology approach was employed to elucidate the mechanism of SN compounds against PCa. For intersection analysis, overlapping proteins between the predicted substance targets and the PCa-related proteome were identified. To build a robust Protein–Protein Interaction (PPI) network, UniProt IDs of the overlapping proteins were imported into Cytoscape (v3.10.1) using the stringApp (v2.2.0). A confidence score cutoff of 0.4 was applied to retrieve interactions from the STRING database. For network integration, the bipartite compound-target network was merged with the STRING-derived PPI network using the Union merge tool in Cytoscape to create a comprehensive systems-level architecture. To assess centrality measures, top candidate proteins were selected based on network topology measures, including degree centrality, to identify hub nodes critical to the SN-target-PCa interactome.

#### 4.2.3. Functional and Pathway Enrichment Analysis

To characterize the biological implications of the identified targets, functional enrichment was performed using the STRING Enrichment designer within Cytoscape. To examine Gene Ontology (GO): Enrichment was categorized into Biological Process (BP), Cellular Component (CC), and Molecular Function (MF). For pathway analysis, KEGG pathway enrichment was conducted to identify significantly modulated signaling circuits.

#### 4.2.4. Molecular Docking

Molecular docking was performed using the Genetic Optimization for Ligand Docking (GOLD) software, GOLD Suite v5.3 [63], to investigate the binding interactions of gallic and ellagic acids with selected cancer-related protein targets. The three-dimensional crystal structures of the proteins were obtained from the Protein Data Bank (PDB) [64] including AKT1 (PDB ID: 4GV1) [65] and CDK2 (PDB ID: 1AQ1) [66]. These targets were selected because they play important roles in cancer-related pathways such as cell proliferation, survival signaling, and cell cycle regulation. Prior to docking, the protein structures were prepared by removing crystallographic water molecules and unnecessary heteroatoms while retaining the relevant protein chains and co-crystallized ligands to define the binding region by Discovery Studio Visualizer program (BIOVIA Discovery Studio 2021) [67]. The binding sites were defined based on the position of the co-crystallized ligand and previously reported active-site residues. The gold_kinase_VS template in GOLD was used for the docking protocol because it is specifically optimized for virtual screening of kinase inhibitors and is suitable for ATP-binding kinases such as AKT1 and CDK2. The three-dimensional structure of gallic and ellagic acids was prepared prior to docking by Gaussian program [68], and ligand flexibility was allowed during the docking process. The GOLD genetic algorithm was employed to generate multiple ligand conformations within the binding pocket, with 500 docking runs performed for each ligand to ensure comprehensive sampling of potential binding poses and improve docking reliability. The resulting docking poses were ranked according to the scoring function implemented in the selected template, and the best binding pose was selected based on docking score. 

### 4.3. In Vitro Experiments

#### 4.3.1. Cell Culture

Human prostate cancer DU145 and PC-3 cells were obtained from Assoc. Prof. Dr. Supachai Yodkeeree (Department of Biochemistry, Faculty of Medicine, Chiang Mai University, Thailand). Cells were cultured in Roswell Park Memorial Institute (RPMI) 1640 and contain 10% fetal bovine serum, 100 U/mL penicillin, and 100 mg/mL streptomycin. Approval number for biosafety in this study is CMUIBC02003/2569.

Peripheral blood mononuclear cells (PBMCs) were prepared from a buffy coat of volunteers at the Blood Bank Unit at Maharaj Nakorn Chiang Mai Hospital, which is affiliated with the Faculty of Medicine at Chiang Mai University, using Histopaque-1077, following the density gradient centrifugation standard protocol. All experiments involving human PBMCs must first be approved by the Institutional Review Board and the Research Ethical Committee of the Faculty of Medicine at Chiang Mai University (Study code: FAC-MED-2569-0048, Research ID: 0048, approval date 16 March 2026). RPMI-1640 medium supplemented with similar DU145 and PC-3 cell lines was used to cultivate isolated PBMCs. Cells were incubated at 37 °C in a 5% CO_2_ environment and prepared for subsequent experiments. Donors were selected based on the criteria established by the Blood Bank Unit [69].

#### 4.3.2. Cytotoxicity Assay

DU145, PC-3, and PBMC cells were subjected to treatment with the SN extract at various concentrations (for prostate cancer: 25, 50, 100, 200, and 400 µg/mL; for PBMC: 50, 100, 200, 500, and 1000 µg/mL) for durations of 24 and 48 h. The cytotoxicity of treated cells was determined using SRB assay. First, cells were seeded at a density of 3.0 × 10^3^ cells or an appropriate number of cells per well in a 96-well plate and incubated overnight at 37 °C in a 5% CO_2_ environment. Then, cells were exposed to varying concentrations of the extract for 24 and 48 h. After treatment, cells were evaluated using the SRB test according to the established technique [70]. The absorbance at 510 nm was measured using a microplate reader (BioTek, Winooski, VT, USA). Then, the 20% and 50% inhibitory concentrations (IC_20_ and IC_50_, respectively) were examined and applied for further experiments. The selective index (SI) of DU145 and PC-3 treated cells was calculated and compared with normal human cells to identify the cytotoxic selectivity of SN extract [19].

#### 4.3.3. Colony Formation Assay

DU145 and PC-3 cell lines were seeded in 6-well plates. After 80% confluence, cells were treated with SN extracts in several concentrations, including 0, 25, 50, 100, 200, and 300 μg/mL for 48 h. After that, the medium was replaced with fresh DMEM containing 10% FBS, and cells were incubated at 37 °C for 10 days. Colonies were fixed with methanol and stained with 0.5% crystal violet. Colonies containing more than 50 cells were quantified using ImageJ software version 1.54g (NIH, Bethesda, MD, USD). Colony images were captured at 2.5× magnification with a 5 × 5 montage using the Lionheart FX Automated Microscope (BioTek, Winooski, VT, USA) and quantified using Gen5 Image Prime software version 3.05.11 (BioTek, Winooski, VT, USA).

#### 4.3.4. Cell Cycle Assay

DU145 and PC-3 cells were plated in 6-well plates and allowed to attach for 48 h before being exposed to different concentrations of SN extracts. After 48 h of treatment, the cells were collected, fixed overnight in 70% ethanol, and stained with propidium iodide (PI) to assess cell cycle distribution. Analysis of cell cycle phases was carried out using a CytoFLEX flow cytometer (Beckman Coulter, La Brea, CA, USA). Representative cell-cycle histograms were analyzed using EasyFlowQ (version 1.5; EasyFlow, an open-source GUI implemented in Python 3.10.4) [71]. Each experiment was conducted in triplicate and repeated independently three times [19,72].

#### 4.3.5. Western Blotting

Cells treated with SN extract for 48 h were lysed with a RIPA buffer and the protein concentration was measured by Bradford assay kit, as described in a previous study [73]. The protein expression was performed using Western blotting. Protein samples will be run on SDS-PAGE and transferred onto nitrocellulose membranes. Then, 5% skim milk diluted in 0.1% Tween-TBS was used to block non-specific binding on the membrane for an hour at room temperature. The membrane was incubated with the individual-specific primary antibodies for cell survival and proliferation, including p-Akt (Cat. No. #9272, Cell Signaling Technology, Beverly, MA, USA), Akt (Cat. No. #9272, Cell Signaling Technology, Beverly, MA, USA), CDK2 (Cat. No. ab32147, Abcam, Cambridge, MA, USA), CDK4 (Cat. No. ab199728, Abcam, Cambridge, MA, USA), and β-actin (Cat. No. ab6276, Abcam, Cambridge, MA, USA) overnight at 4 °C. After that, membranes were washed and incubated with peroxidase-labeled secondary antibodies (Thermo Fisher Scientific, Waltham, MA, USA) for 2 h at room temperature. Finally, membranes were incubated with the SuperSignal West Pico Chemiluminescent Substrate (Thermo Fisher Scientific, USA). The intensity of each band was analyzed using ImageJ (NIH image) analysis software.

#### 4.3.6. Statistical Analysis

One-way ANOVA followed by Tukey’s post hoc analysis was used for in vitro experiments in this study. Data were illustrated as mean ± SD. A *p*-value < 0.05 was considered as significant. For silico approach, a Benjamini–Hochberg adjusted *p*-value < 0.05 was set as the threshold for significance. Enrichment results were visualized using dot plots to highlight the most relevant mechanisms of action.

## 5. Conclusions

SN represents a promising source of bioactive compounds with potential relevance for prostate cancer management. Its phytochemical constituents are associated with anti-proliferative activity, particularly through the regulation of cell-cycle progression and cell survival pathways. Together with the network pharmacology and docking data suggesting that gallic acid and ellagic acid can interact with proliferation-related targets such as AKT1 and CDK2. Therefore, SN extract exerts selectivity to reduce cancer, supporting its potential development as a therapeutic strategy for advanced and hormone-independent prostate cancer. However, further in vivo and clinical investigations are warranted to validate its efficacy, safety, and translational potential.

## Figures and Tables

**Table 1:** Cytotoxicity (IC_20_ and IC_50_) of SN against prostate cancer cells (DU145 and PC3) and normal PBMC at 24 and 48 h, and the corresponding selectivity indices.

Time		24 h	48 h
DU145:	IC_20_ (µg/mL)	293.97 ± 8.15	167.46 ± 14.12
	IC_50_ (µg/mL)	627.97 ± 46.49	465.70 ± 26.71
PC3:	IC_20_ (µg/mL)	250.96 ± 36.30	223.47 ± 36.17
	IC_50_ (µg/mL)	672.17 ± 97.20	457.00 ± 22.68
PBMC *	IC_20_ (µg/mL)	>1000	>1000
	IC_50_ (µg/mL)	>1000	>1000
		Selectivity index (SI) **	
DU145		>1.59	>2.15
PC3		>1.49	>2.19

* PBMC: Peripheral blood mononuclear cells. ** Selectivity index (SI) is the ratio between IC_50_ of PBMCs and that of prostate cancer cells. IC: inhibitory concentration.

## Data Availability

The original contributions presented in this study are included in the article/Supplementary Material. Further inquiries can be directed to the corresponding author.

## Supplementary Materials

The following supporting information can be downloaded at: https://www.mdpi.com/article/10.3390/ijms27135977/s1.
