# Supplementary material for: Network Pharmacology and Molecular Docking of Syzygium nervosum Extracts on Antiproliferative Effect in Prostate Cancer
Source: Int J Mol Sci. 2026 Jul 3;27(13):5977. doi: 10.3390/ijms27135977 (PMC13362457; doi:10.3390/ijms27135977)
Supplement: Supplementary file 1 [file ijms-27-05977-s001.zip › ijms-4388383-supplementary.pdf]

Network Pharmacology and Molecular Docking of *Syzygium nervosum* Extracts on Antiproliferative Effect in Prostate Cancer

Supplementary Materials

Supplementary Figure 1. Redocking validation of AKT1 and CDK2 docking. The top-scoring poses of the redocked ligands were superimposed with the corresponding co-crystallized ligand conformations.

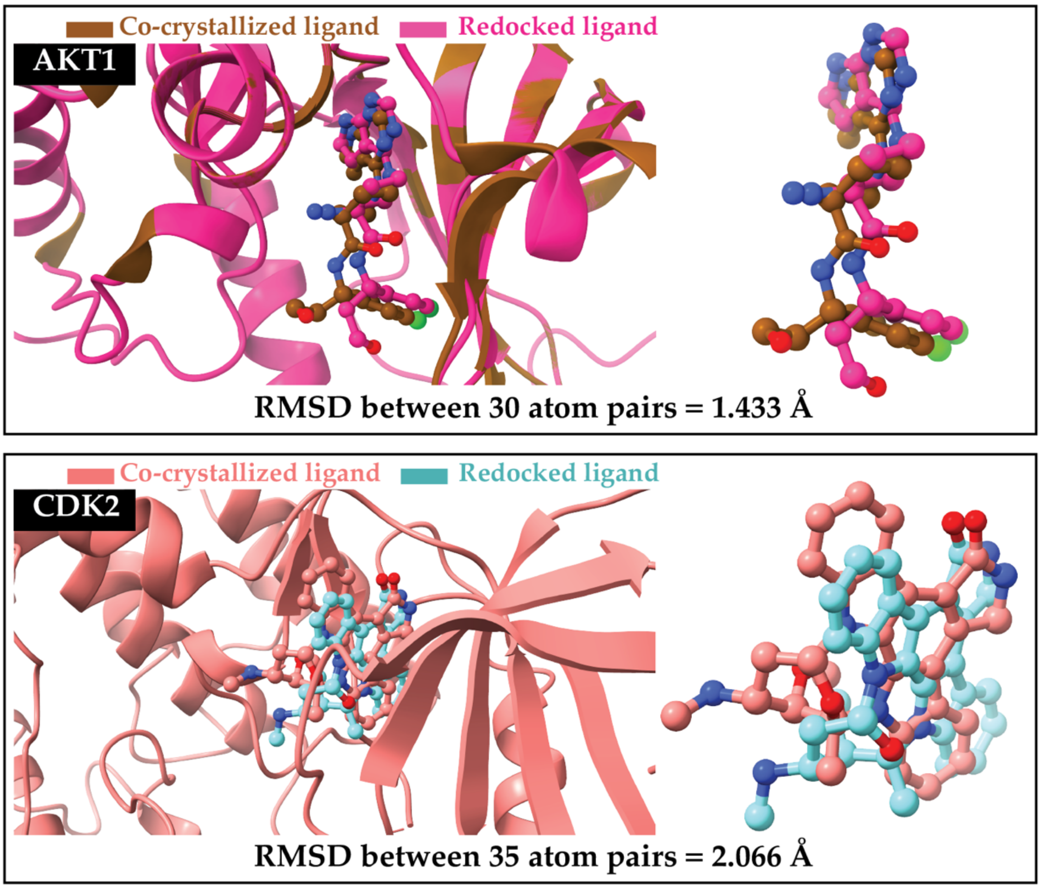

Figure S1. The redocked ligand poses were superimposed with the original co-crystallized ligand conformations, yielding RMSD values of 1.433 Å for AKT1 and 2.066 Å for CDK2. These results indicate that the docking protocol reasonably reproduced the experimental ligand-binding poses. We also clarified that docking results should be considered supportive computational evidence only and that further experimental assays are required to confirm direct target engagement.

To validate the docking protocol, the co-crystallized ligands were removed from the AKT1 and CDK2 structures and redocked into their original binding sites. The top-scoring redocked poses were then superimposed onto the experimentally determined ligand conformations. The calculated RMSD values were 1.433 Å for AKT1 and 2.066 Å for CDK2, indicating that the docking protocol reproduced the experimental ligand orientations with reasonable accuracy (Figure S1). However, docking results should be considered supportive computational evidence only, and further experimental assays are required to confirm direct target engagement."
